# The moderating role of workplace spirituality on the effect of organizational justice on job satisfaction

**DOI:** 10.3389/fpsyg.2024.1360913

**Published:** 2024-08-02

**Authors:** Esin Ertemsir, Yasemin Bal, Ayşe Demirhan, Özgür Kökalan

**Affiliations:** ^1^Department of Business Administration, Yıldız Technical University, Istanbul, Türkiye; ^2^Department of Business Administration, Istanbul Sabahattin Zaim University, Istanbul, Türkiye

**Keywords:** organizational justice, job satisfaction, workplace spirituality, moderating role, aviation industry

## Abstract

The study’s goal is to investigate the moderating effect of the workplace spirituality of employee on the relationship between their organization’s justice perception and job satisfaction. The study included a sample of 360 employees from two Turkish airline companies. The findings show a relationship between organizational justice and job satisfaction. The study also finds that workplace spirituality moderates the effect of organizational justice on job satisfaction. Employees with high workplace spirituality are more satisfied than those with low workplace spirituality. The study is important, especially in examining the moderator role of workplace spirituality between these two variables, and fills a gap in the literature. Empirical data of Turkiye’s two largest airline companies, which aim to become and remain competitive in the aviation industry, were shared. This research-based approach provides guidelines for this industry on the effects of workplace spirituality on job satisfaction and organizational justice.

## Introduction

Outside of the religious perspective, workplace spirituality (WPS) concerns the well-being of employees, organizations, and communities. WPS has been a research area that has attracted attention from many disciplines recently. WPS, seen as one of the most important factors in coping with workplace stress, has become one of the topics considered relevant by practitioners and the academic field in recent years. The combination of the words “workplace” and “spirituality” in the 1980s caused a contradiction. However, this situation has created an impersonal community awareness by highlighting “meaningful work and harmony with workplace values.”

There are various definitions of WPS in the literature. Cavanagh (1999) defined this concept as “the desire to find the ultimate purpose in life and live accordingly.” Ashmos and Duchon (2000) defined spirituality in the workplace as “the recognition of an inner life that is fostered and nourished by meaningful work that takes place in the context of community.” Giacalone and Jurkiewicz (2003) defined WPS as “a framework of organizational values evidenced in a culture that encourages employees’ experience of transcendence throughout the work process, facilitating their sense of connection with others in ways that provide feelings of wholeness and joy.” WPS is an employee’s effort to find their value in life and align this value with the values of the workplace (Mitroff and Denton, 1999). What is expressed here is that the employee finds his job meaningful and can make positive evaluations about the purpose and goal of his career. Community awareness, by catching the synergy of the employee with other employees, is to act in a common spirituality and solidarity. Finally, harmony with work and business values means that the employee has common values with the work environment and increases his contribution to the environment (Pelenk and Acaray, 2020).

Workplace spirituality in terms of individual and organizational outputs is among the expected features for organizations in today’s business environment. WPS is slightly different from spirituality itself. The boundaries of WPS are drawn with the workplace of an organization. According to Ashmos and Duchon (2000), workplace spirituality is not just related to religion or forcing people to accept a particular belief system. Instead, WPS is about employees who perceive themselves as spiritual beings whose souls must be nurtured in the work environment. Corner (2009) did not equate spirituality with religion or confine it to religion. Spirituality, he maintained, is any fulfillment, pleasure, understanding, or connection to or with people or a philosophy. According to several research studies, spirituality may be any sense of dedication, understanding, and satisfaction, not just toward religion but also toward people and oneself. WPS focuses more on tolerance, patience, a sense of interconnectedness, purpose, and acceptability of mind to the organization’s norms (Hassan et al., 2016). The practice of WPS gives purpose and meaning to work and offers employees a better sense of work, leading to improved organizational performance. Properly managing WPS can control individual behavior in the workplace (Eliyana and Sridadi, 2020). When employees bring their spiritual side to work, they can be more creative, leading to higher performance (Turner, 1999). One of the important reasons why WPS has gained importance today is the difficulties experienced by employees in ensuring work-life balance. This is why employers today increasingly encourage WPS to increase employee loyalty and morale (Bhaskar and Mishra, 2019). Thus, WPS is an important tool in improving employee happiness and satisfaction.

While there are studies examining the association between various variables and WPS, studies have yet to be found in the literature that looks at the function of WPS as a moderator in the relationship between job satisfaction and organizational justice. In response to the pandemic-related increase in resignations and searches for meaningful employment, spirituality research has focused on themes such as job satisfaction (Kumar et al., 2022; Ghayas et al., 2023; Suwono et al., 2023; Sarpong et al., 2024) and organizational justice (Hwang and Yi, 2021). While several recent studies have investigated the moderator (Pelenk and Acaray, 2020; Hilton et al., 2024) or mediator effect (Jayakumar and Vinodkumar, 2023; Sode and Chenji, 2024) of WPS in the relationships between various variables, its moderator role in the link between OJ and JS has not been investigated. As long as employees perceive insecurity and organizational injustice, they cannot be satisfied with their jobs. Workplace Spirituality begins to be questioned in all its aspects in this case. However, spirituality might have a moderating impact once the connection between job satisfaction and perceptions of justice is established. Finding purpose in our work, upholding amicable and cooperative working relationships, and connecting with others and our inner selves can contribute to job satisfaction (Naz et al., 2017).

This study tries to fill this gap in the literature by including empirical data from Turkiye’s aviation sector. The study’s goal is to investigate the moderating effect of the workplace spirituality of employee on the relationship between their organization’s justice perception and job satisfaction.

Overall, in response to the gaps mentioned above, this study addresses the following research questions:

*RQ1. Is organizational justice (OJ) positively associated with job satisfaction (JS) in the aviation industry?*

*RQ2. Does workplace spirituality (WPS) strengthen the positive relationship between OJ and JS in the aviation industry?*

The paper’s first section provides a literature review on workplace spirituality, organizational justice, and job satisfaction, as well as studies that address these three concepts. The second section covers the research methods, analysis, and findings and then discusses the practical and managerial implications, limitations, and recommendations for future work. Finally, an overall review is provided in the conclusion.

## Literature review

### Workplace spirituality

#### Definitions and components of WPS

There are various definitions of the subject in the literature. The terms “organizational spirituality,” “workplace spirituality,” and “management, spirituality, and religion” were all used to describe the phenomenon in the early 1990s (Kamoche and Pinnington, 2012). Hill et al. (2000) argued that spirituality is an essential function of religion. Ashmos and Duchon (2000) identified three WPS components: inner life, meaningful work, and community. Milliman et al. (2003) define WPS as a complex and multifaceted construct that includes three basic dimensions. The purpose of one’s job at the individual level is to have a “sense of community” at the group level and to be “in line with the organization’s values and mission” at the organizational level. WPS is the state of the spiritual well-being of an individual in the work environment in which employees feel peaceful with the organizational structure (Altaf and Awan, 2011; Pawar, 2016). This can include different factors affecting an individual’s life or job satisfaction. The phrase “workplace spirituality” is used in this article and concentrates on the literature on spirituality driven from the top and administered like corporate culture.

#### Impact of WPS on employees

Krishnakumar and Neck (2002) stated that promoting WPS can benefit employees and organizations. It is seen that WPS is important, especially in increasing efficiency, creativity, and organizational commitment (OC) and JS. Sarpong et al. (2024) attempted to find a positive impact between WPS and JP (job performance). According to Sarmad et al. (2018), workplace spirituality and Islamic business ethics significantly positively impact employee performance, including increased job satisfaction. Providing a spiritual environment and maintaining the spirituality of the workplace not only ensures a satisfied employee but also results in high productivity, morale, and increased competition. Thus, WPS can improve happiness, well-being, and job attitudes in JS and OC (Asutay et al., 2021).

For this reason, WPS can be seen as one of the prominent strategies for organizations to have sustainable competitive advantage (Al-Hosaini and Sofian, 2015). There will be better working conditions and satisfaction when the organization has a high WPS (Garcia-Zamor, 2003; Altaf and Awan, 2011). WPS may provide employees with additional resources to cope with job-related challenges while encouraging personal and professional growth and fulfillment by leading them to acquire the strength to deal with difficult situations at work (Bhaskar and Mishra, 2019). Employees can handle stressful environments more efficiently and overcome difficulties with the support of WPS.

#### Organizational outcomes of WPS

WPS helps make work meaningful and integrate people into society. For this reason, one’s spirituality in the workplace not only results in increased performance but also allows the person to get away from stress and conflict, making him a good person (Duchon and Plowman, 2005). High WPS reduces the effect of harmful emotional labor on employee well-being, while low WPS increases the impact of harmful emotional labor on employee well-being (Zou and Dahling, 2017). WPS practice has been shown to influence employees’ emotions and spirit, resulting in higher dedication, satisfaction, creativity, and emotional stability, which can contribute to the organization’s high performance (Krishnakumar and Neck, 2002). WPS encompasses acts of compassion, empathy, support for others, and confidence in oneself and others. Employees and organizations should apply their values more correctly by integrating them into their jobs. Hence, actions that meet spiritual requirements must consciously develop a spiritual work environment (Hassan et al., 2016; Bella et al., 2021). Fry (2003) states that future organizations must recognize WPS. If the organization understands the purpose and meaning of employees’ values and lives, it will meet their needs (Ashmos and Duchon, 2000). Employees will be more devoted to the company and content with their lives due to organizational practices that enable them to sense a high degree of meaning in work, membership, and inner life, which are the focal sub-dimensions of WPS (Jeon and Choi, 2021). Thus, harmony will be achieved between the employees and the organization’s values and lives.

#### WPS researches

Some studies include the literature’s relationships between WPS and different variables (Genty et al., 2017; Charzynska et al., 2021; Rehman et al., 2021). Noor and Arif (2011) investigated the relationship between WPS and JS. Kumar and Kumar (2014) found that WPS had a moderating effect between stress and a healthy work environment. Hassan et al. (2016) showed that WPS had a significantly positive relationship with trust, and trust significantly mediated the impact of WPS on JS. Zou and Dahling (2017) showed that WPS regulates the effects of harmful emotional labor on employee well-being. Genty et al. (2017) found a statistically significant positive relationship between WPS and normative OC. Awada et al. (2020) found that mental and emotional well-being had a positive moderator effect on employee happiness and employee performance. In other words, it showed that increasing the employees’ moral and emotional health levels would positively affect employee happiness and performance. Corso et al. (2020) found that WPS was related to higher positive affectivity, resilience, and self-efficacy. Bella et al. (2021) proposed a model for spiritual factors in the workplace. This paradigm can also help people comprehend that their mental health, like their body and mind, needs to be taken care of. This model’s primary use is in workplace design, specifically in how moral human aspects in HRM may be employed in strategic planning. Hanafi et al. (2021) found that WPS positively and significantly impacted organizational performance.

Nguwasen (2021) searched the effect of WPS on employee commitment and found a direct and positive significant effect. WPS and perceived organizational support (OS) have a favorable impact on business control and organizational citizenship behavior (OCB), according to Adıgüzel et al. (2021). Furthermore, the effect of WPS on task control and OCB was mediated by perceived OS. Bibi et al. (2021) recommended that top managers deploy WPS to develop employee OCB. All these studies show how effective WPS is in improving employee productivity and business performance. Some recent studies focus on the influence of organizational justice and workplace spirituality on mental health (Iman et al., 2024) and occupational health (Chirico et al., 2023). The latest study has shown a connection between occupational health psychology and workplace spirituality through yoga and other spiritual practices. Literature on WPS has advanced significantly in recent years, but additional study is required to pinpoint relevant moderators and mediators (Saxena and Prasad, 2023). The importance of investigating WPS as a moderator is among the aims of this study.

### Organizational justice

Organizational justice (OJ) is one of the important factors in increasing employee satisfaction and commitment. Organizational justice scholars seek to understand how individuals perceive justice and what they consider. Therefore, organizational justice prioritizes subjective fairness above objective justice. According to the organizational justice theory, justice is socially produced and subjective (Adamovic, 2023). The employees must perceive the organization’s management, processes, and practices relatively. The employees’ fair perception of internal practices, procedures, and processes is of great importance for employee satisfaction and productivity. OJ is one of the powerful factors affecting JS. OJ has a strong influence on employee behaviors. If a person in a workplace perceives fair pricing and fair promotion opportunities, they will be satisfied with their job (Robbins, 1998). When organizations treat their employees fairly, employees develop a positive feeling and attitude toward the organization (Imran et al., 2015). Employees treated relatively can be happier and more satisfied, making their organizations more effective (Lazarus, 2000; Bakotic, 2016).

Fairness is the cornerstone of Equity Theory (Adams, 1965), which has been widely used in organizational behavior (Chen et al., 2015). OJ is founded on the notion of justice or fairness. Theoretically, three types of OJ are widely mentioned in organizational research literature: distributive justice (DJ), procedural justice (PJ), and interactional justice (IJ; Karkoulian et al., 2016), each of which can contribute to the variance in JS either individually or collectively. To begin, DJ refers to an individual’s perception of the fairness of the outcomes received by the organization. When evaluating DJ, comparisons of employee inputs (effort) and organizational outcomes (salary, appreciation, performance rating, and so on) are utilized as the evaluation base (Whitman et al., 2012). As viewed by the employees, the perceived objectivity of the ruling process is PJ (Colquitt, 2001). PJ is achievable when choices are made via fair mechanisms, such as a complete reward system (Folger and Greenberg, 1985). Employees tend to report less procedural fairness when favoritism is seen because promotions and prizes are allocated based on personal relationships rather than individual achievement (Sonnentag, 2012). Finally, IJ is concerned with how workers perceive OJ regarding communication and interpersonal treatment (Ambrose et al., 2002). It conveys their viewpoint on how fair organizational authority is handled while making organizational choices (Palaiologos et al., 2011). The presence of PJ and DJ in the company and HR policies such as fair wages and benefits, training and career development, and objective performance evaluation impact employees’ perspectives of organizational cooperation. In this study, the authors analyzed OJ justice under a single dimension.

### Job satisfaction, OJ, and WPS

Locke (1969) defined job satisfaction (JS) as “a function of the perceived relationship between what a person wants from her job and what she perceives to be what she offers “. JS is the employees’ attitudes toward their jobs and the institutions they work for. In other words, JS is an employee’s emotional reaction to work based on a contrast between real and intended job results (Mosadeghrad, 2003). A person with high satisfaction shows a positive attitude toward work. Conversely, people who are dissatisfied with their job will have a negative attitude toward their job (Robbins, 2006).

Also, it is known that there is an important relationship between OJ and JS. JS increases when OJ increases; when OJ perception decreases, JS decreases (Lawson et al., 2009). Al-Zu’bi (2010) found a strong connection between OJ and JS. Employee JS was dependent on the managers’ OJ. Ozel and Bayraktar (2018) showed that OJ positively affected the JS of employees. OJ is one of the determining factors of JS.

For this reason, organizations that aim to provide JS to their employees should take effective steps to ensure OJ (Çetinsöz and Turhan, 2016). Rovenska’s research findings (2018) revealed that PJ, DJ, and IJ were significant predictors of JS. Afridi and Baloch (2018) showed that OJ was positively associated with JS.

The employee who adds a spiritual meaning to his work first enriches his inner world, increasing JS by gaining physical, cognitive, and spiritual well-being (Mitroff and Denton, 1999). Therefore, there is an important link between JS and WPS. WPS makes communication, justice, and management skills important among employees. In this context, WPS leads to correct and high-quality communication and the understanding and love of employees. Employees with WPS try to understand external customers by revealing their emotional identities, and managers also gain their trust by showing interest in employees (Gockel, 2004). Several researchers have found that WPS favors JS (Gupta et al., 2014; Hassan et al., 2016; Sapta et al., 2021). When WPS rises, JS rises with it. In one of the first studies, Milliman et al. (2003) revealed that WPS is strongly associated with employees’ job attitudes, such as JS and work engagement. According to Jurkiewicz and Giacalone (2004) values framework of WPS, the perception of justice rises when employees are treated equally and fairly evaluated; rewards and/or punishments are assigned relatively and unbiasedly; on the other hand, dishonesty, faithlessness, and wrongful or biased judgments detract from the justice value. The overlap of the results obtained by the employee from her job with her needs and value judgments provides JS. This shows the importance of the relationship between WPS, OJ, and JS.

According to Walt and Klerk (2014) study, there is an association between WPS and JS, and they propose more confirmatory empirical investigations using more complex statistical analysis to confirm the link between WPS and JS. Korkmaz and Menge (2018) investigated the role of WPS on OC and found that WPS had a positive effect on OC. Iyer and Deshmukh (2018) found that WPS moderated the relationship between role ambiguity and JS of nurses. Bhaskar and Mishra (2019) investigated WPS’ mediating and moderating effect in its relationship with perceived OS, career satisfaction, and turnover intentions. WPS improved employee career satisfaction while drastically lowering turnover intentions by modulating the link between perceived OS and turnover intention. Pelenk and Acaray (2020) revealed that WPS positively affected JS and moderated the relationship between excessive workload and JS.

In the literature, it is possible to come across many studies investigating WPS’s mediator and moderator role on different variables. One of the links addressed by several studies is the moderator effect of WPS on work overload and JS (Altaf and Awan, 2011; Saeed et al., 2017). These two research conclusions contradict each other. While Altaf and Awan (2011) discovered that WPS played a moderating function in the link between workload and JS, Saeed et al. (2017) found that WPS did not play a moderating role in the association between workload and JS. Another variable connected with WPS is leadership style. Transformational leadership (McKee et al., 2011) and spiritual leadership (SL) are widely investigated related to WPS (Sani et al., 2018; Astuti and Haryani, 2020; Sapta et al., 2021). McKee et al. (2011) explored the mediation of transformational leadership between employee mental well-being and WPS’ community dimension rather than WPS as a single dimension. According to Sani et al. (2018), WPS modulates the influence of SL on OCB. According to Astuti and Haryani (2020), WPS has a favorable and substantial influence on emotional commitments, and SL also has a mediating effect on emotional commitment. Sapta et al. (2021) state that WPS mediates SL’s impact on OC. Intention to quit the job, one of the indicators used to understand the peacefulness and pleasure of people at work, has also been a concept that interests us about its relationship with WPS. Bhaskar and Mishra (2019) investigated the role of WPS in mediating and moderating the significant relationship between perceived OS, career satisfaction, and turnover intentions. WPS has been shown to considerably lower employee turnover intentions while boosting employee career satisfaction by altering the relationship between perceived OS and turnover intention. Charzynska et al. (2021) investigated whether the WPS regulates the direct and indirect impacts of quantitative and emotional job demands on teachers’ turnover intention. Hwang and Yi (2021) evaluated the effects of WPS and organizational justice on workers’ intentions to leave their jobs. Sode and Chenji (2024) identified workplace spirituality as a partial mediator in the relationship between self-transcendence and innovative work behavior, effectively nurturing employees’ creative capacities.

#### Research gaps and hypotheses

While the moderator or mediator effect of WPS in the relations between different variables has been examined in several recent studies (Pelenk and Acaray, 2020; Pariyanti et al., 2021; Hilton et al., 2024), its moderator role in the relationship between OJ and JS has not been examined. Pelenk and Acaray (2020) investigated the effect of work overload on job satisfaction and the moderating effect of workplace spirituality. It was found that “workplace spirituality positively affects job satisfaction” and “workplace spirituality has a moderating role in the relationship between excessive workload and job satisfaction” in this study, which involved Turkish employees. Their paradigm needed to include organizational justice. Hilton et al. (2024) searched if workplace spirituality is an effective moderator in the association between ethical leadership, transformational leadership, and employees’ job involvement. Their research provides findings to bridge the gap in the WPS literature on leadership. They confirmed that workplace spirituality moderately affected leadership and job involvement associations. However, job satisfaction needed to be added to their model. Pariyanti et al. (2021) searched to determine whether or not Islamic WPS (IWPS) acts as a moderator in the relationship between OJ, JS, and Workplace Deviant Behavior (WDB). According to the findings, IWPS is a buffer between OJ and workplace deviance. When an organization encourages Islamic spirituality at work, workers show appreciation because they feel their efforts will be rewarded in the afterlife. Workers belonging to Islamic workplace spirituality will consider their work a genuine and honest form of prayer. Their moderation model was the most similar to ours. However, Pariyanti and colleagues concentrated on Islamic WPS and included organizational injustice and workplace deviance factors in addition to JS in their model. A broader sense of spirituality, not limited to religion, is lacking here. Prior studies show that WPS strongly predicts JS (Saeed et al., 2017). In addition to these confirmed benefits of WPS, this study focuses on whether WPS strengthens the positive relationship between OJ and JS, which is missing in the literature.

Specific service sectors, including aviation, faced more difficult working circumstances than others following the pandemic’s worldwide lockdown (Yüksek et al., 2022; Zeynel, 2023). This study tries to fill this gap in the literature by including empirical data from Turkiye’s aviation sector. The employees in the aviation sector were under greater pressure than many professional groups to prevent passengers from contracting the disease while performing their duties during the COVID-19 pandemic.

A study by Kim Quy et al. (2024) on the aviation sector in Vietnam, a country with a collectivist culture comparable to Turkey, found that in the post-COVID era, employees will find it simpler to align with organizational goals if the organization supports workplace spirituality since it will encourage them to be less self-centered and more linked to the broader picture the organization portrays. In the context of the epidemic, they were required to adhere to tight aviation safety requirements and always be on duty. Therefore, building a culture of equity, trust, and holistic well-being is advised. In this context, as a professional group that does not have the opportunity to work remotely during the pandemic period and in an environment where working together as a group and supporting one another is critical, decisions are made based on what is best for the group overall (Kim Quy et al., 2023) it was important to investigate the workplace spirituality, job satisfaction, and organizational justice perceptions of aviation industry employees while the effects of the pandemic were continuing.

Even though the literature on WPS has grown recently, Sharma and Kumra (2020) and Saxena and Prasad (2023) noted that further research is necessary to determine the moderating and mediating impacts of WPS and OJ. Their research findings recommended that WPS may be a strong motivator that directly affects related variables, and there is a need to explore the relationship of the WPS dimensions with other work attitudes. Building on the gap proposed by these researchers, the authors determined a need to test not only potential mediation but also moderation effects between the OJ relationship and employee outcomes such as JS, trust, and perceived autonomy in the workplace; therefore, we use WPS as a moderator to narrow the suggested research gap. In this direction, the following hypotheses have been developed.

*H*1: Organizational justice (OJ) is positively associated with job satisfaction (JS).

*H*2: Workplace spirituality (WPS) is positively associated with job satisfaction (JS).

*H*3: Workplace spirituality (WPS) moderates the effect of organizational justice (OJ) on job satisfaction (JS).

## Materials and methods

### Participants and procedure

This study is a cross-sectional study. Data were collected during January and May 2021 from employees working in two Turkish airline companies in Turkiye. According to the data of the Turkish Ministry of Transportation, the number of employees in the Turkish Aviation sector is 53,885 as of the end of 2021.^1^ Random and snowball sampling were used in the research. In the first step of the data collection process, consent was taken from the participants. In this process, the questionnaire was formed and distributed via Google Forms. The survey was delivered to the managers working at the airline company via e-mail, and these managers made sure that the questionnaire reached the participants in the WhatsApp groups they established within the workplace. Some participants sent it to their colleagues working in airline companies. Only those willing to participate were included in the study. When the data was examined, no participant stated that they did not want to participate in the research. It is still being determined how many people in total the questionnaire used within the scope of the study reached. Since no participant stated that they did not want to answer the questionnaire, the non-response rate could not be calculated within the scope of the research.

The questionnaire was gathered from respondents at two different times with a two-month time lag to decrease common method bias and avoid the biasing effects of occasional factors (Podsakoff et al., 2012; Matthews et al., 2014). Especially in behavioral sciences, the Common Method Variance problem is most encountered. The distribution between these two variables consists of measuring each dependent and independent variable together and responding at the same speed. In this case, the estimated effects may be negatively affected by Common Method Bias. In particular, using surveys can cause serious difficulties due to the risk of common methods. Common method bias is another variable presented by the estimates to be made. In this case, what exists among the variables is systematically increased. However, there are up-to-date answers to the explanations about temporary moods (Tehseen et al., 2017). One of the solutions suggested for CMV by Podsakoff et al. (2003) is to separate the measurements of the variables of the measurements temporally, methodologically, or psychologically. In this position, it is a practice to place measurements at a different time interval when obtaining data on variables. In addition, great importance will be given to conveying the respondents’ behavior, attitudes, and perceptions with minimal errors in terms of the results to be obtained in terms of management and theoretical inferences. The questionnaire consisted of 4 parts. In the first part, the participants were asked six questions to determine their socio-demographic characteristics. The questions about OJ, WPS, and JS were asked in the second, third, and last parts. When the sample number is evaluated based on a 95% confidence interval and a 5.2% margin of error, it is at least 356. At the end of this process, 360 data were gathered from the contributors.

PowerPower analysis was performed with the GPower program to determine a sufficient number of samples to determine sufficient number of samples. Since multiple linear regression analysis will be used to test the hypotheses, valid parameters were selected for this analysis in the GPower program. The study was carried out by entering the parameters into the program so that the number of independent variables was 3, the effect size (f^2) was 0.05, the probability of type one error (α) was 0.05, and the test power (1-β) was 0.95. According to the test results, the minimum number of samples required was determined to be 262, which shows that the sample amount reached is reasonably sufficient for the analyses.

Demographic information about the participants is given in Table 1.

**Table 1:** Demographical characteristics of the participants.

		Frequency	Percent (%)	Valid percent (%)	Cumulative percent (%)
Gender	Female	173	47.4	48.1	48.1
	Male	187	51.2	51.9	100.0
	Total	360	100.0	100.0	
Marital Status	Married	242	67.2	67.2	67.2
	Single	118	32.8	32.8	100.0
	Total	360	100.0	100.0	
Age	25 and below	18	4.9	5.0	5.0
	26–30	134	36.7	37.2	42.2
	31–40	162	44.4	45.0	87.2
	41–50	46	12.6	12.8	100.0
	Total	360	100.0	100.0	
Education Level	High School	15	4.1	4.1	4.1
	Associate Degree	15	4.1	4.1	8.2
	Bachelor Degree	203	55.6	55.6	63.8
	Graduate	127	36.2	36.2	100.0
	Total	360	100.0	100.0	

The research sample consisted of 187 men (51.2%) and 173 women (48.1%). Their ages ranged from 25 to 50. Their average airline industry work experience was 8.6 years (SD 6.11). 91.8% had at least a bachelor’s degree. It is seen that the gender distribution of the participants is close to each other; almost all of them have a high level of education and are young and middle-aged.

### Measure

Three different scales called the “Organizational Justice Scale” (OJS), “Workplace Spirituality Scale” WPSS, and “Job Satisfaction Scale” JSS were used in the study. The OJS was developed by Niehoff and Moorman (1993) and adopted in Turkish by Taşçıoğlu (2010). It consisted of 20 items and three subscales. Sample items are “My work schedule is fair” and “The manager makes job decisions in a biased manner.” As a result of confirmatory factor analysis (CFA), the fit indexes were as follows: x^2^/df = 4.267, GFI = 0.902, AGFI = 0.877, CFI = 0.916, and RMSEA = 0.088. Therefore, the model fit was determined as an acceptable level (Hair et al., 1998). Cronbach’s alpha for OJS was 0.829.

The WPSS was developed by Kinjerski and Skrypnek (2006). It consisted of 18 items and four subscales called “Engaging Work,” “Sense of Community,” “Spiritual Connection,” and “Mystical Experience.” It is adapted in Turkish with 16 items and four subscales. Sample items are “I can find meaning or purpose at work,” “I receive inspiration or guidance from a Higher Power about my work,” and “I experience a connection with a greater source that has a positive effect on my work.” As a result, CFA’s fit indexes were as follows: x^2^/pdf = 3.112, GFI = 0.908, AGFI = 0.891, CFI = 0.914, and RMSEA = 0.082. Therefore, the model fit was determined as an acceptable level (Hair et al., 1998). Cronbach’s alpha for WPS was 0.774.

The JSS used in the study was developed by Brayfield and Rothe (1951), shortened by Judge et al. (1998), and adopted into Turkish by Başol and Çömlekçi (2020). It consisted of 5 items. Sample items are “I feel fairly satisfied with my present job” and “I find real enjoyment in my work. As a result, CFA’s fit indexes were as follows: x^2^/df = 2.772, GFI = 0.934, AGFI = 0.904, CFI = 0.928, and RMSEA = 0.056. Therefore, the model fit was very good (Hair et al., 1998). Cronbach’s alpha for JSS was 0.807. The overall Cronbach alpha value of the model is 0.889.

All items in three different scales were rated with a 5-point Likert scale ranging from 1 (strongly disagree) to 5 (strongly agree).

In our analysis, gender, education level, age, and job experiences were taken as demographic variables.

The data was analyzed using SPSS 25 and AMOS 25 programs. All scale items are given in Appendix 1.

## Results

### Descriptive statistics and correlations

Descriptive statistics about variables and correlations among variables are given in Table 2. In this process, the normality for each variable was first checked. It was seen that all variables are normally distributed.

**Table 2:** The descriptive statistics of the data.

	Average	Std. Dev	OJ	WPS	JS
Organizational Justice (OJ)	3.52	0.583	1		
Workplace Spirituality (WPS)	3.53	1.036	−0.044	1	
Job Satisfaction (JS)	3.90	0.613	0.540**	0.746**	1
Gender	1.52	0.500	−0.015	0.046	0.022
Education	2.66	0.764	0.033	−0.059	−0.089
Age	3.23	0.715	0.050	−0.025	0.063
Work Experience	3.71	1.180	−0.051	−0.042	−0.002

*N* = 360. **p < 0.01.

The mean of the OJ, WPS, and JS were 3.52 ± 0.58, 3.53 ± 1.03, and 3.90 ± 0.61, respectively. According to the independent sample t-test of OJ, WPS, and JS levels, it was seen that there are no differences according to gender (*t* = 0.281, *p* = 0.77; *t* = −0.862, *p* = 0.38; *t* = −0.419, *p* = 0.67). The one-way ANOVA test of OJ, WPS and JS level shown that there are no differences in OJ, WPS and JS level according to education level of respondents (*F* = 0.195, *p* = 0.90; *F* = 1.427, *p* = 0.23; *F* = 1.240, *p* = 0.29).

Correlation analysis showed that there is a positive correlation between OJ and JS (*r* = 0.540, *p* < 0.01) and WPS and JS (*r* = 0.746, *p* < 0.01). It was also determined that there is no statistically significant correlation between OJ and WPS (*r* = −0.044, *p* > 0.05).

### Hypotheses testing

#### Measurement model

**Figure 1:**
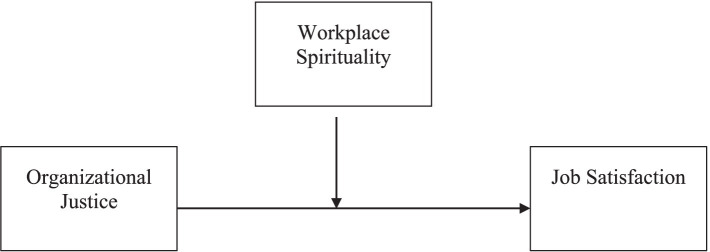
Theoretical model.

All moderator variable analysis steps were required with SPSS and AMOS programs. First, variables used within the scope of the analysis were standardized with the help of SPSS. Then, a third variable (intersection) was obtained by multiplying the OJ and WPS variables. With this data set in SPSS, moderator variable analysis was performed with multiple regression in AMOS. The theoretical model is given in Figure 1.

Using measuring instruments like discriminant validity, composite reliability, and convergent validity, measurement models assess the validity and reliability to produce accurate computations. According to the study results, all items’ loading factor values are greater than 0.5, so they are declared valid. All research indicators’ AVE values are more than 0.5, and all MSV values of research indicators are less than the AVE value, so it satisfies the convergent validity requirement. Both Cronbach’s alpha and the composite reliability values of research indicators are more than 0.7; thus, they met the reliability test criteria (Hair et al., 1998). All this information is given in Table 3.

**Table 3:** Discriminant validity, composite reliability, and convergent validity.

Construct	Items	Factor loading	AVE	MSV	CR	*C. alpha*
Workplace spirituality	WS1S	0.886	0.56	0.42	0.78	0.78
	WS2S	0.861				
	WS3S	0.933				
	WS4S	0.872				
	WS5S	0.835				
	WS6S	0.830				
	WS7S	0.767				
	WS8S	0.856				
	WS9S	0.933				
	WS10S	0.924				
	WS11S	0.890				
	WS12S	0.894				
	WS13S	0.788				
	WS14S	0.771				
	WS15S	0.890				
	WS16S	0.836				
	WS17S	0.922				
	WS18S	0.864				
Organizational justice	OJ1S	0.770	0.52	0.46	0.81	0.82
	OJ2S	0.773				
	OJ3S	0.702				
	OJ4S	0.893				
	OJ5S	0.739				
	OJ6S	0.766				
	OJ7S	0.800				
	OJ8S	0.826				
	OJ9S	0.792				
	OJ10S	0.789				
	OJ11S	0.822				
	OJ12S	0.736				
	OJ13S	0.729				
	OJ14S	0.759				
	OJ15S	0.846				
	OJ16S	0.834				
	OJ17S	0.764				
	OJ18S	0.866				
	OJ19S	0.752				
	OJ20S	0.730				
Job satisfaction	JS1S	0.882	0.53	0.48	0.79	0.80
	JS2S	0.842				
	JS3S	−0.776				
	JS4S	0.782				
	JS5S	−0.826				

#### Path analysis and moderation

The Path and moderation analysis results are given in Table 4.

**Table 4:** Path and moderating analysis.

Path charts			Path coefficient	Std. error	t statistics	*p*-value	Description
OJ	➔	JS	0.326	0.020	16.588	***	H1 accepted
WPS	➔	JS	0.343	0.028	12.430	***	H2 accepted
OJ*WPS	➔	JS	0.645	0.036	17.763	***	H3 accepted

***p < 0.01.

**Figure 2:**
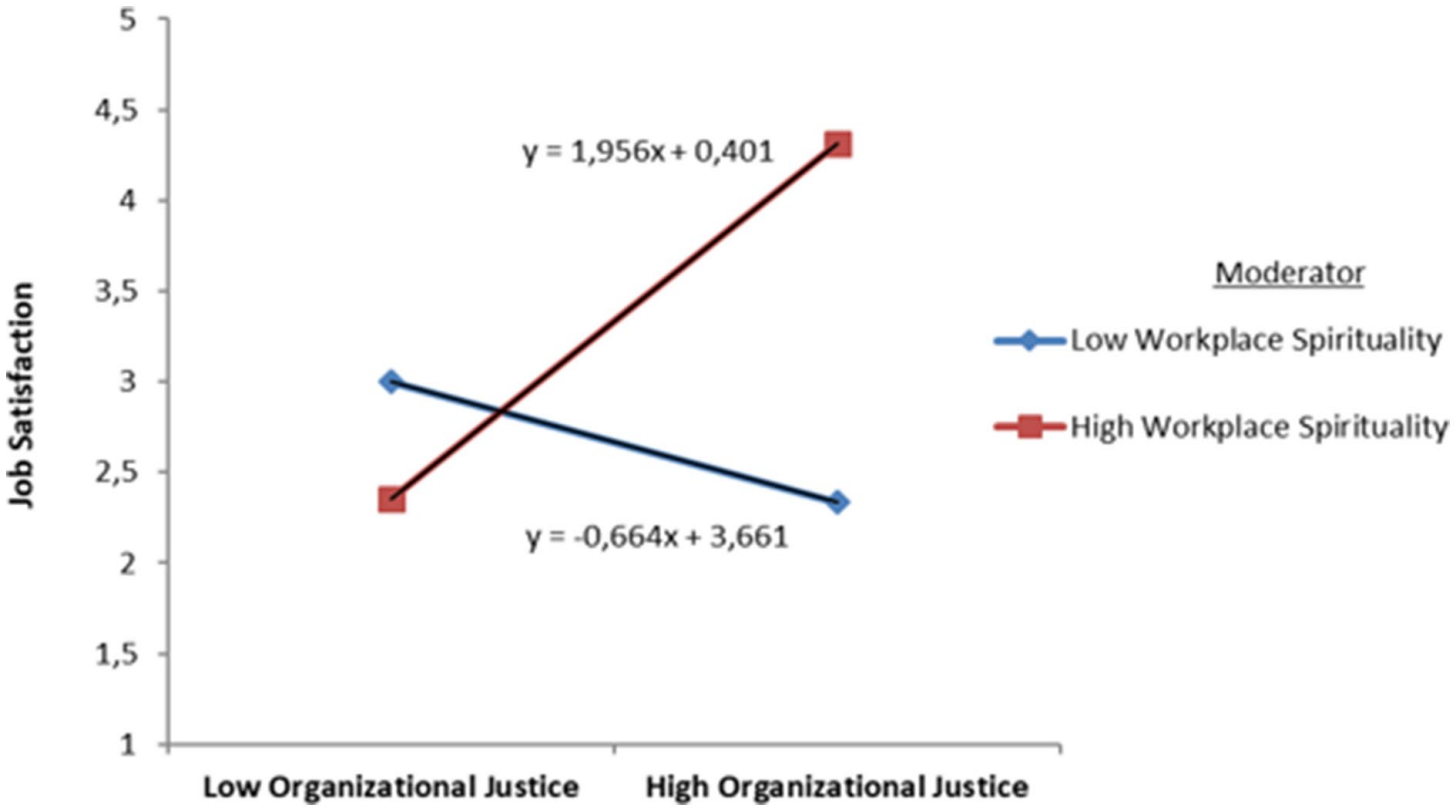
The interaction between OJ and WPS.

H1 suggests that OJ is positively associated with JS. Table 4 represents that OJ is positively associated with JS (*β* = 0.326, *t* = 16.588, SE = 0.020, *p* < 0.01). H2 is supported. H2 suggests that WPS is positively associated with JS. Table 4 represents that WPS is positively associated with JS (*β* = 0.343, *t* = 12.430, SE = 0.028, *p* < 0.01). H2 is supported. H3 suggests that WPS moderates the effect of OJ on JS. Table 4 shows that the interaction term was significant (*β* = 0.645, *t* = 17.763, SE = 0.036, *p* < 0.01). The result indicates that WPS strengthens the positive relationship between OJ and JS. H3 is also supported. Figure 2 shows this relationship.

The predictive power of the model is controlled by the R-Square value. Calculating R -Square value is given in Table 5.

**Table 5:** R-square result.

	R square	R square adjusted
Structural model	0.939	0.921

Table 5 shows that the R – Square value on JS is 0.939. R – Square indicates that independent variables in the structural model influenced JS of 93.9%.

## Discussion, implıcatıons, limitations and future research directions

Research on spirituality has increased since 2020, coinciding with an increased intention to leave work and a desire to find meaning in work observed in corporate life during the pandemic. Specific industries suffered more than others from declining business volume and subsequent job losses. As it slowed down travel. The epidemic brought new and unprecedented challenges to job security in the aviation sector (Yüksek et al., 2022; Zeynel, 2023) as well as the tourism and services sectors (Lee et al., 2023). In this regard, the aviation industry’s data from 2021 -shortly after the pandemic’s general shutdown period- provides important and timely data regarding employees’ job satisfaction, perceived organizational justice, and spirituality. The findings of this study indicate that OJ is positively associated with JS. It is found that there are no differences in OJ, WPS, and JS levels according to the gender and education level of respondents. Previous literature agrees that OJ positively affects JS (Moorman, 1992; Rovenska, 2018; Pariyanti et al., 2021). Furthermore, OJ is stated as a determining factor of JS (Lawson et al., 2009; Afridi and Baloch, 2018).

The study’s second goal was to investigate the moderating effect of the workplace spirituality of employee on the relationship between their organization’s justice perception and job satisfaction. Although the moderator or mediator effect of WPS in the relations between different variables has been examined in the previous studies, its moderator role in the relationship between OJ and JS has not been investigated. The authors use WPS as a moderator variable to fill the gap in the literature on WPS moderation and its effect on OJ on JS. Based on the findings on aviation industry employees, the study finds that workplace spirituality moderates the impact of organizational justice on job satisfaction. Consistent with previous research, this study found that employees’ more robust sense of workplace spirituality strengthens the positive association between their organizational justice perceptions and job satisfaction (Ihsan et al., 2020; Yarım, 2021). Prior studies show that WPS strongly predicts JS (Saeed et al., 2017). As stated by Nurhasan et al. (2023), the higher the value of spirituality in the workplace, such as togetherness and goal alignment, supported by job happiness, the better employees perform. Walt and Klerk (2014) and Adawiyah et al. (2020) proved this relationship by finding a positive relationship between WPS and JS. Also, Belwalkar and Vohra (2016) found relationships between OCB, JS, and WPS. Fanggida et al. (2016) studied the influence of WPS on OC and JS. In addition to these confirmed benefits of WPS, this study found that WPS strengthens the positive relationship between OJ and JS.

### Practical and managerial implications

Regarding the study’s practical and professional consequences, the researchers suggest addressing aviation industry employees with the healing power of WPS and concepts such as sincerity, responsibility, and community involvement. According to Kim Quy et al. (2023), air service providers should emphasize nurturing workplace spirituality in the aviation industry. Workplace spirituality is becoming increasingly important in today’s multinational business world, where ethnic discrimination is on the rise owing to migration policies, and we desire a collaborative culture and diversity management. Employees in the aviation industry constantly have access to people from other cultures, whether as colleagues or passengers. Practices on workplace spirituality help workers better endure various requests and complaints and approach them with a sense of collective existence.

Furthermore, with Turkiye’s collectivist culture with a score of 46 in mind (2024),^2^ aviation employee training should focus on motivating employees to collaborate and achieve collective goals. These behaviors, which foster a sense of community in the workplace, may also serve as a model for other collectivist countries regarding cultural dimensions. To improve job satisfaction, HR training can include a series of short films describing the meaning of work, demonstrating how they add value to the passengers’ journey and contribute to the world. While Hilton et al. (2024) propose a purposeful effort to develop workplace spirituality, qualities like compassion, virtue, purpose, dedication, and belonging are also recommended. According to Elamin and Hayfaa, 2015, Islamic spirituality and social responsibility are positively related to perceived organizational justice, increasing employee happiness and well-being.

In this study, spirituality was discussed beyond religious doctrine. Although data was collected from a Muslim country, the literature review was not limited to Islamic Spirituality. Spirituality was explored outside the confines of spiritual teaching. Although the study did not restrict spirituality to religion, data was obtained mainly from Muslim employees. However, the study’s findings benefit not only Muslim employees but also have universal implications in terms of spiritual and religious doctrine due to the interaction of Muslims and non-Muslims. Such research raises non-Muslims’ understanding of their Muslim coworkers’ spiritual requirements in the workplace (Asutay et al., 2021). In line with the findings of this study, a growing body of empirical evidence has emerged highlighting specific cases in which the implementation of workplace spirituality interventions resulted in tangible improvements in organizational justice and job satisfaction, providing valuable insights and practical guidance for managers looking to improve these critical organizational outcomes. Leaders prioritizing spiritual values, such as servant orientation, diversity, and openness, are likelier to foster positive employee attitudes and productivity. These suggestions for achieving greater job satisfaction and commitment can provide practical guidance to managers.

### Limitations and future research directions

This study was carried out with some limitations. In the study’s data collection process, it was tried to reach as many people working in airline companies as possible. However, the number of participants reached constitutes the most critical limitation of this research. Although a sufficient number of participants were reached for analysis due to G Power analysis, the number of participants should be increased to more reliable results in further studies; the selection of a small sample size can strongly impact the generalizability of the study findings. Small sample sizes frequently result in overestimating effect sizes, inadequate replication, and restricted applicability of findings to larger populations. Additionally, within the scope of this research, how many people the survey used in the study reached and how many people responded need to be clarified. In other words, this study could not calculate the response rate. The lack of clarity in this information prevents us from seeing the difference between those who took the survey and answered it and those who took it and did not answer it. This is another sampling limitation of the research. In other words, this study’s inability to calculate the response rate is a significant limitation. It prevents understanding potential non-response bias, which could affect the study’s validity. To improve the response rate in future research, the scales used in the study may be short, survey timing can be rearranged, a different distribution method can be used, different incentives can be given to the participants, or friendly reminders can be sent to respondents. Third, this research was conducted on aviation industry employees, and its results may need to be generalizable to different industries. Also, this finding opens up further research possibilities for extending the research to various sectors. Another recommendation is to investigate the relationships between WPS, OJ, and JS subdimensions, as the authors regarded all aspects within their umbrella term and accepted them as a whole dimension. This study is a cross-sectional study. The data was collected in 2021, and the results obtained in this study may differ now. This and all similar studies have this kind of limitation. Longitudinal studies are also advised while the impacts of this time are still being felt. The findings of this study can also be considered from the perspective of the Great Resignation Phenomenon triggered by the pandemic. During this period, employees’ search for meaning in their work increased, and its effects on job satisfaction became more controversial (Tessema et al., 2022). Finally, the participants of this study were only Turkish employees. In future studies, more comprehensive and comparative studies on WPS can be done on employees of other nationalities. If a broader discussion is desired, data collected from other countries may provide an opportunity to discuss cultural dimensions and variances in the interpretation of spirituality across religions.

## Conclusion

This study contributes to the literature by deepening our theoretical understanding of workplace spirituality and providing empirical evidence. By offering WPS as a moderator variable, the authors aim to fill in the gap in previous research on WPS moderation and the impact of OJ on JS. Based on the study findings, an employee with high WPS levels is more satisfied than an employee with less WPS. It reveals that WPS and OJ should be among the issues managers should pay attention to, especially in increasing employee performance and JS. All future organizations should consider WPS to enhance employees’ well-being. When an organization perceives the purpose and meaning of employees’ lives, then it will meet the employee’s needs and expectations in a better way. In particular, managers should consider WPS so that the employees can cope with the negative organizational factors in their organization more easily and be productive. At this point, it is seen as one of the prominent factors that managers develop applications in a way that will ensure WPS. As a result, fostering WPS can improve JS.

## Data availability statement

The raw data supporting the conclusions of this article will be made available by the authors, without undue reservation.

## Ethics statement

Ethical review and approval were not required for the study on human participants in accordance with the local legislation and institutional requirements. The patients/participants provided their written informed consent to participate in this study.

## Author contributions

EE: Conceptualization, Data curation, Investigation, Supervision, Validation, Writing – original draft. YB: Conceptualization, Data curation, Validation, Visualization, Writing – original draft. AD: Conceptualization, Data curation, Methodology, Validation, Writing – original draft. ÖK: Supervision, Writing – review & editing.

## Footnotes

^1^ https://www.uab.gov.tr/uploads/pages/havacilik/03-sektor-raporlari-havayolu.pdf

^2^ hofstede-insights.com
