# Supplementary material for: The moderating role of workplace spirituality on the effect of organizational justice on job satisfaction
Source: Front Psychol. 2024 Aug 2;15:1360913. doi: 10.3389/fpsyg.2024.1360913 (PMC11327939; doi:10.3389/fpsyg.2024.1360913)
Supplement: Supplementary file 1 [file Data_Sheet_1.docx]

**Appendix**

**Questionnaire**

1. Gender a) Female b) Male
2. Marital status a) Single b) Married
3. Age …. year/s
4. Education Level a) High School b) Associate Degree

c) Bachelor Degree d) Graduate

1. Work experience …. year/s

**Job Satisfaction Scale - Brayfield and Hve Rothe (1951)**

| No | Items | Definitely disagree | Disagree | Neither Agree nor Disagree | Agree | Definitely agree |
| --- | --- | --- | --- | --- | --- | --- |
| 1 | I feel fairly well satisfied with my present job. |  |  |  |  |  |
| 2 | Most days, I am enthusiastic about my work. |  |  |  |  |  |
| 3 | Each day of work seems like it will never end. (reverse-scored) |  |  |  |  |  |
| 4 | I find real enjoyment in my work. |  |  |  |  |  |
| 5 | I consider my job rather unpleasant. (reverse-scored) |  |  |  |  |  |

**Spirit At Work Scale- Kinjerski and Skrypnek (2006)**

| No | Items | Definitely disagree | Disagree | Neither Agree nor Disagree | Agree | Definitely agree |
| --- | --- | --- | --- | --- | --- | --- |
| 1 | I experience a match between the requirements of my work and my values, beliefs and behaviors. |  |  |  |  |  |
| 2 | I am able to find meaning or purpose at work. |  |  |  |  |  |
| 3 | I am passionate about my work |  |  |  |  |  |
| 4 | I am fulfilling my calling through my work. |  |  |  |  |  |
| 5 | I have a sense of personal mission in life, which my work helps me to fulfill. |  |  |  |  |  |
| 6 | I feel grateful to to be involved in work like mine. |  |  |  |  |  |
| 7 | At the moment, I am right where I want to be at work. |  |  |  |  |  |
| 8 | At times, I experience a “high” at work. |  |  |  |  |  |
| 9 | I have moments at work in which I have no sense of time or space. |  |  |  |  |  |
| 10 | At moments, I experience complete joy and ecstasy at work. |  |  |  |  |  |
| 11 | I experience moments at work where everything is blissful. |  |  |  |  |  |
| 12 | At times, I experience an energy or vitality at work that is difficult to describe. |  |  |  |  |  |
| 13 | My spiritual beliefs play an important role in everyday decisions that I make at work. |  |  |  |  |  |
| 14 | I receive inspiration or guidance from a Higher Power |  |  |  |  |  |
| 15 | I experience a connection with a greater source that has a positive effect on my work. |  |  |  |  |  |
| 16 | I feel like I am part of “a community” at work. |  |  |  |  |  |
| 17 | I experience a real sense of trust and personal connection with my coworkers. |  |  |  |  |  |
| 18 | I share a strong sense of purpose and meaning with my coworkers about our work |  |  |  |  |  |

**Organizational Justice - Niehoff and Moorman (1993)**

| No | Items | Definitely disagree | Disagree | Neither Agree nor Disagree | Agree | Definitely agree |
| --- | --- | --- | --- | --- | --- | --- |
| 1 | My work schedule is fair |  |  |  |  |  |
| 2 | I think that my level of pay is fair |  |  |  |  |  |
| 3 | I consider my workload to be quite fair |  |  |  |  |  |
| 4 | Overall, the rewards I receive here are quite fair |  |  |  |  |  |
| 5 | I feel that my job responsibilities are fair |  |  |  |  |  |
| 6 | Job decisions are made by the manager in an unbiased manner |  |  |  |  |  |
| 7 | My manager makes sure that all employee concerns are heard before job decisions are made |  |  |  |  |  |
| 8 | To make job decisions, my manager collects accurate and complete information |  |  |  |  |  |
| 9 | My manager clarifies decisions and provides additional information when requested by employees |  |  |  |  |  |
| 10 | All job decisions are applied consistently across all affected employees |  |  |  |  |  |
| 11 | Employees are allowed to challenge or appeal job decisions made by the manager |  |  |  |  |  |
| 12 | When decisions are made about my job, the manager treats me with kindness and consideration |  |  |  |  |  |
| 13 | When decisions are made about my job, the manager treats me with respect and dignity |  |  |  |  |  |
| 14 | When decisions are made about my job, the manager is sensitive to my personal needs |  |  |  |  |  |
| 15 | When decisions are made about my job, the manager deals with me in a truthful manner |  |  |  |  |  |
| 16 | When decisions are made about my job, the manager shows concern for my rights as an employee |  |  |  |  |  |
| 17 | Concerning decisions made about my job, the manager discusses the implications of the decisions with me |  |  |  |  |  |
| 18 | The manager offers adequate justification for decisions made about my job |  |  |  |  |  |
| 19 | When making decisions about my job, the manager offers explanations that make sense to me |  |  |  |  |  |
| 20 | My manager explains very clearly any decision made about my job |  |  |  |  |  |
